# Speech Emotion Recognition in Mental Health: Systematic Review of Voice-Based Applications

**DOI:** 10.2196/74260

**Published:** 2025-09-30

**Authors:** Eric Jordan, Raphaël Terrisse, Valeria Lucarini, Motasem Alrahabi, Marie-Odile Krebs, Julien Desclés, Christophe Lemey

**Affiliations:** 1 ObTIC Sorbonne Université Paris France; 2 URCI Mental Health Department Brest Medical University Hospital Brest France; 3 EA 7479 SPURBO Université de Bretagne Occidentale Brest France; 4 Université Paris Cité, Institute of Psychiatry and Neuroscience of Paris (IPNP), INSERM U1266, team “Pathophysiology of Psychiatric disorders :Development and Vulnerability” Paris France; 5 GHU-Paris Psychiatrie et Neurosciences, Hôpital Sainte Anne, Evaluation, Prevention and Therapeutic Innovation Department, F-75014 Paris France; 6 IMT Atlantique, Lab-STICC, UMR CNRS 6285, F-29238 Brest France

**Keywords:** affective computing, machine learning, mental health, psychology, psychiatry, speech emotion recognition, voice

## Abstract

**Background:**

The field of speech emotion recognition (SER) encompasses a wide variety of approaches, with artificial intelligence technologies providing improvements in recent years. In the domain of mental health, the links between individuals’ emotional states and pathological diagnoses are of particular interest.

**Objective:**

This study aimed to investigate the performance of tools combining SER and artificial intelligence approaches with a view to their use within clinical contexts and to determine the extent to which SER technologies have already been applied within clinical contexts.

**Methods:**

The review includes studies applied to speech (audio) signals for a select set of pathologies or disorders and only includes those studies that evaluate diagnostic performance using machine learning performance metrics or statistical correlation measures. The PubMed, IEEE Xplore, arXiv, and ScienceDirect databases were queried as recently as February 2025. The Quality Assessment of Diagnostic Accuracy Studies tool was used to measure the risk of bias.

**Results:**

A total of 14 articles were included in the final review. The included papers addressed suicide risk (3/14, 21%), depression (8/14, 57%), and psychotic disorders (3/14, 21%).

**Conclusions:**

SER technologies are mostly used indirectly in mental health research and in a wide variety of ways, including different architectures, datasets, and pathologies. This diversity makes a direct assessment of the technology challenging. Nonetheless, promising results are obtained in various studies that attempt to diagnose patients based on either indirect or direct results from SER models. These results highlight the potential for this technology to be used within a clinical setting. Future work should focus on how clinicians can use these technologies collaboratively.

**Trial Registration:**

PROSPERO CRD420251006669; https://www.crd.york.ac.uk/PROSPERO/view/CRD420251006669

## Introduction

### Background

Emotions play a pivotal role in human interaction and communication, influencing various aspects of social exchange, decision-making, and overall well-being. While emotions can be expressed through multiple modalities, including facial expressions, body language, and gestures, human speech remains one of the most prominent and accessible channels for conveying emotional states.

Intonation, rhythm, pitch, and other acoustic features of speech convey subtle emotional cues, reflecting an individual’s psychological well-being [1,2]. In recent years, there has been increasing interest in leveraging advancements in machine learning (ML) to analyze and interpret emotional cues from speech, a field known as speech emotion recognition (SER). Interest in the automated detection of mental disorders through vocal features is growing, particularly in the context of mental health assessment and monitoring. The ability to automatically analyze and interpret emotional cues from speech offers several advantages for improving patient care, enabling early detection of mental health issues, and enhancing the overall health care experience [3-6].

To better understand the foundations of SER and its evolving landscape, it is important to consider its historical development, theoretical models, and methodological challenges.

### History of SER

The field of SER originated in the 1990s, when it emerged as a subsection within the field of speech processing. Initial work approached the task by extracting acoustic features from recordings and then performing statistical analysis using various algorithms to derive correlations between the extracted features and the emotional state of the speaker [7]. The original papers published on the topic suggested that the automatic detection of emotions could be applied in the context of human-machine interaction.

These initial publications led to further interest within the field and prompted questions such as the modeling of emotions and the contrast between acted and nonacted emotions. The choice of how to represent emotions is a nontrivial issue and has historically required coordination with the field of psychology to ensure the model used is both compatible with the machines being used for automatic recognition and consistent with the psychological literature on emotions [8].

**Figure 1:**
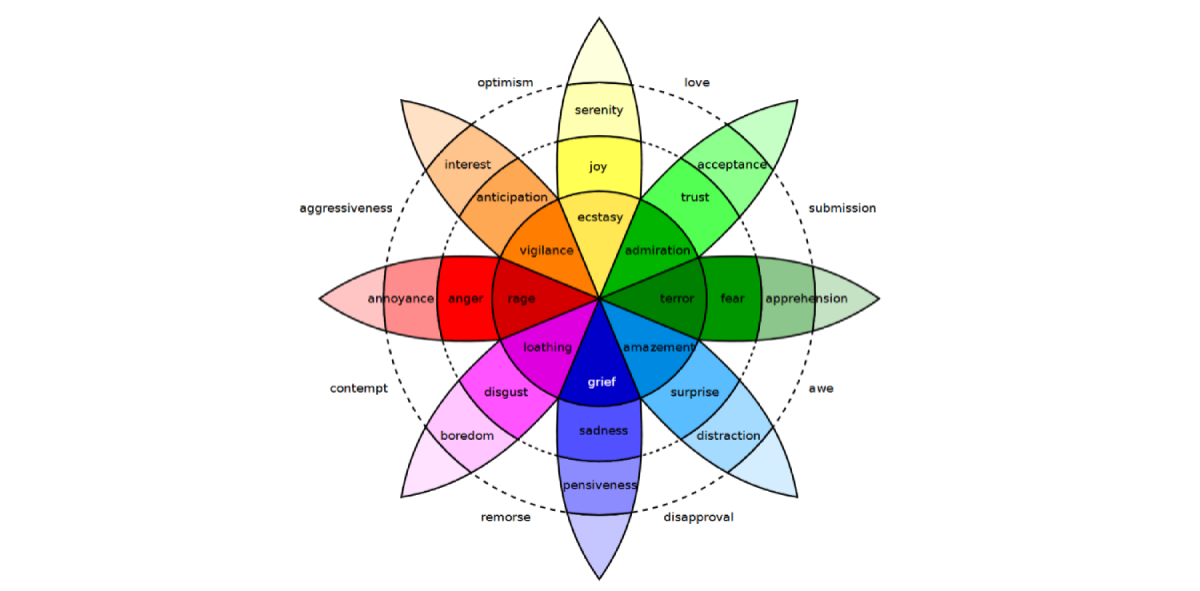
The wheel of emotions proposed by Plutchik.

The models typically used fall into 2 groups. First, *categorical models* represent emotions as separate “classes” (eg, happy, sad, and angry). The “big six” model proposed by Ekman et al [9] is a well-known example, encompassing happiness, sadness, anger, fear, disgust, and surprise. A more nuanced extension of this categorical approach is the model developed by Plutchik [10], which expands the core emotions by incorporating trust and anticipation, forming an 8–primary-emotion framework. The model also introduces the concept of emotional intensity and relationships between emotions, visualized in the “wheel of emotions” (Figure 1), where primary emotions blend to form more complex emotional states. Subsequent research has often adapted emotional classifications by adding new categories, removing some, or merging similar emotions into a single class.

**Figure 2:**
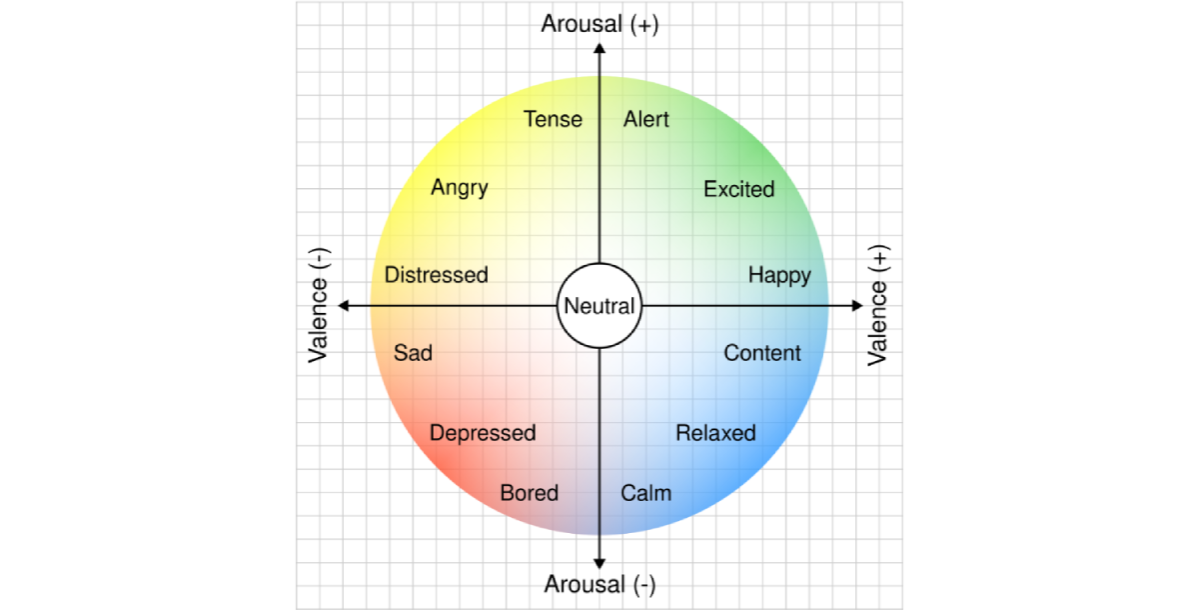
The circumplex model of emotion with its 2 axes: valence and arousal.

Second, *dimensional* or *continuous* models interpret emotions as degrees of some underlying features. These models typically involve plotting emotions on 2 or 3 axes, the most common of which is the 2-dimensional model of arousal and valence [11]. In this paradigm, valence reflects the positive or negative quality of emotion. Happiness has high valence, while sadness has low valence. Arousal reflects the level of physiological activation or intensity associated with emotion. For example, surprise and anger typically have high arousal, while sadness and contentment often have low arousal (Figure 2).

Much of the existing work in SER predominantly relies on the 6-scale model developed by Ekman and the 8-scale model developed by Plutchik [10], as they offer well-defined categories that facilitate annotation and classification [12]. In contrast, dimensional approaches, which conceptualize emotions along continuous axes, remain comparatively underexplored. However, the dimensional perspective provides a more nuanced framework for capturing emotional complexity and extends beyond speech processing to psychopathology.

### From Initial Works to Challenges

By the late 2000s, the field of SER had garnered more widespread interest in speech processing, with the development of a wide variety of feature extraction methods and ML algorithms. However, these advancements gave rise to several problems, most notably a lack of comparability among results.

Facing these problems in the late 2000s, researchers in the field initiated a series of SER challenges, including the INTERSPEECH 2009 Emotion Challenge [13]. These competitions aimed to standardize key aspects of the SER process, such as datasets, feature extraction methods, and algorithms, to facilitate the generation of comparable results within a unified context. One significant outcome of these efforts was the development of the open-source Speech and Music Interpretation by Large-Space Extraction (openSMILE) tool for feature extraction [14].

### Arrival of Neural Networks to SER

With the introduction of convolutional neural networks in automatic speech recognition in 2012 [15] through their application to spectrograms, these algorithms were quickly adopted for use in SER contexts [16].

Following trends in both natural language processing (NLP) and automatic speech recognition, various neural network (NN) architectures were also applied. These methods ranged from training NN models directly on SER-related data to training on large quantities of unrelated speech data and then fine-tuning on SER datasets.

### Convergence Between SER and Mental Health

Just as emotions can be viewed from a categorical or dimensional perspective, these approaches can also be applied to mental health disorders. Traditional diagnostic classifications, for example, the *Diagnostic and Statistical Manual of Mental Disorders* (*DSM*) and the *International Classification of Diseases*, adopt a categorical view of mental disorders, which has long served as the standard reference, despite this approach not always being relevant to clinical issues. However, these models come with some limitations when applied to clinical issues. The shift toward viewing disorders as existing along a continuum rather than within rigid diagnostic categories is exemplified by the research domain criteria (RDoC) classification in psychiatry, developed by the National Institute of Mental Health. The RDoC marks a significant evolution in the approach to mental disorders by focusing on biological, cognitive, and behavioral criteria rather than just clinical symptoms. Thus, the RDoC classification proposes a dimensional approach that aims to investigate the underlying processes of mental disorders by addressing transnosographic domains, such as emotion, cognition, or motivation [17]. This shift from categorical classification to a dimensional approach leads to the study of psychiatric disorders as spectrums of dysfunction rather than as distinct entities.

### Operational Definition of Direct and Indirect SER

For the purposes of this review, we distinguish between direct and indirect SER approaches as applied in mental health research. This distinction refers to whether emotion is explicitly modeled and analyzed as a task in its own right or implicitly captured through emotionally relevant features. Direct SER refers to approaches in which emotion recognition is an explicit step in the analysis pipeline. This typically involves training or fine-tuning models on emotion-labeled datasets or applying pretrained SER systems to detect emotional states in speech recordings. The detected emotions are then analyzed in relation to mental health conditions. Indirect SER, by contrast, involves the use of features, models, or techniques that capture emotional characteristics of speech without explicitly recognizing or classifying emotions. For example, acoustic features used in SER may be extracted and used in models for mental health classification, even though emotion labels are not used at any point. These features carry emotional information, but emotion itself is not the target variable.

Given the role of emotion recognition in identifying psychiatric disorders and the increasing interest in applying SER within clinical psychiatry, where vocal emotional cues offer a noninvasive and objective window into patients’ mental states, this study examines the specific role of emotion recognition in identifying psychiatric disorders. To provide context, we review the advancements, applications, and challenges of SER in health care, highlighting its potential in mental health monitoring, suicide prevention, and diagnosis of mood and psychotic disorders. This review emphasizes the advantages of SER, including its noninvasive nature, objective assessment capabilities, and potential for automated analysis, while addressing key challenges, such as the need for diverse datasets, interpretability of ML models, and generalization across populations.

This paper is structured as follows: in the first section, we introduce the history and advancements in SER. Subsequently, we review the available SER datasets, followed by an overview of existing applications of speech- and emotion-related technologies in clinical settings. Finally, we discuss current challenges, limitations, and prospects for collaboration between the fields of SER and mental health.

## Methods

### Preferred Reporting Items for Systematic Reviews and Meta-Analyses Review

Recognizing and analyzing emotions is an essential tool for the clinician. The fields of psychiatry and psychology have long recognized that learning and mastering this skill is at the very core of the diagnostic and therapeutic process, not only for health care professionals but also for patients. However, this clinical skill is neither infallible nor sufficient for systematic precision diagnosis. Several studies have focused on the automated recognition of emotions using a variety of computational techniques. What can these new techniques offer to clinicians in the analysis and interpretation of emotions?

To answer these questions, we conducted a systematic review of literature following the PRISMA (Preferred Reporting Items for Systematic Reviews and Meta-Analyses) guidelines, registering our review in the PROSPERO system (1006669). This review aims to cover studies adopting an acoustic- or speech-related analysis of mental health questions, with a particular focus on works involving an analysis of the emotional aspect of speech when studies paid attention to the emotional dimension.

### Inclusion and Exclusion Criteria

The inclusion criteria for this review are described in Textbox 1.

**Study inclusion and exclusion criteria.**

**Inclusion criteria**

- Studies containing an analysis of speech (ie, audio) signal
- Studies containing either a direct or indirect emotion recognition component. Indirect components include analyses that could be interpreted from an emotional perspective, for example, the use of the open-source Speech and Music Interpretation by Large-Space Extraction (openSMILE) feature sets widely used in speech emotion recognition tasks
- Speech data was derived from a clinical context

**Exclusion criteria**

- Audio was only analyzed in conjunction with another modality (eg, text).
- No diagnosis or prognosis aspect was included, for example, analyzing emotions in isolation without a correlation to patient conditions or outcomes.
- The pathology examined could be classified as a neurological disorder rather than a mental health disorder, for example, Alzheimer disease.
- The study was a review with no experimental component.

### Search Strategy and Screening Process

Database queries were performed using the PubMed, IEEE Xplore, arXiv, and ScienceDirect databases up until February 2025, with the following keyword search: (“emotion recognition” OR “affective computing” OR “emotional analysis”) AND (“psychiatry” OR “psychology”) AND (“speech” OR “voice”).

During the screening process, 2 authors applied the eligibility criteria and selected the studies to be included in the systematic review. In case of doubt, it was established that the article would be submitted to the rest of the review group, and no such cases were encountered.

An initial screening based on the title and abstract of the search results was performed, removing any articles that did not meet the inclusion criteria.

From this initial screening, the remaining articles were assessed to determine whether their design included a direct evaluation of the models’ performance in a diagnostic task, measured by either ML metrics (*F*_1_-score, accuracy, and area under the curve [AUC]) or statistical correlation measures. Most articles excluded in this step did not include an audio component, did not evaluate the clinical diagnostic performance of their model, or applied their model to patients whose pathology was outside the scope of this review (sometimes analyzing only healthy control participants).

After filtering based on these criteria, a total of 14 studies were retained. The Quality Assessment of Diagnostic Accuracy Studies–2 framework was used to estimate the risk of bias in these selected studies.

The Results section is organized in subsections to enhance the reader’s understanding, particularly regarding the methodological context of SER. First, we provide an overview of the methods used within SER and several existing databases. We then present the results of the systematic review.

## Results

### PRISMA Review Results

**Figure 3:**
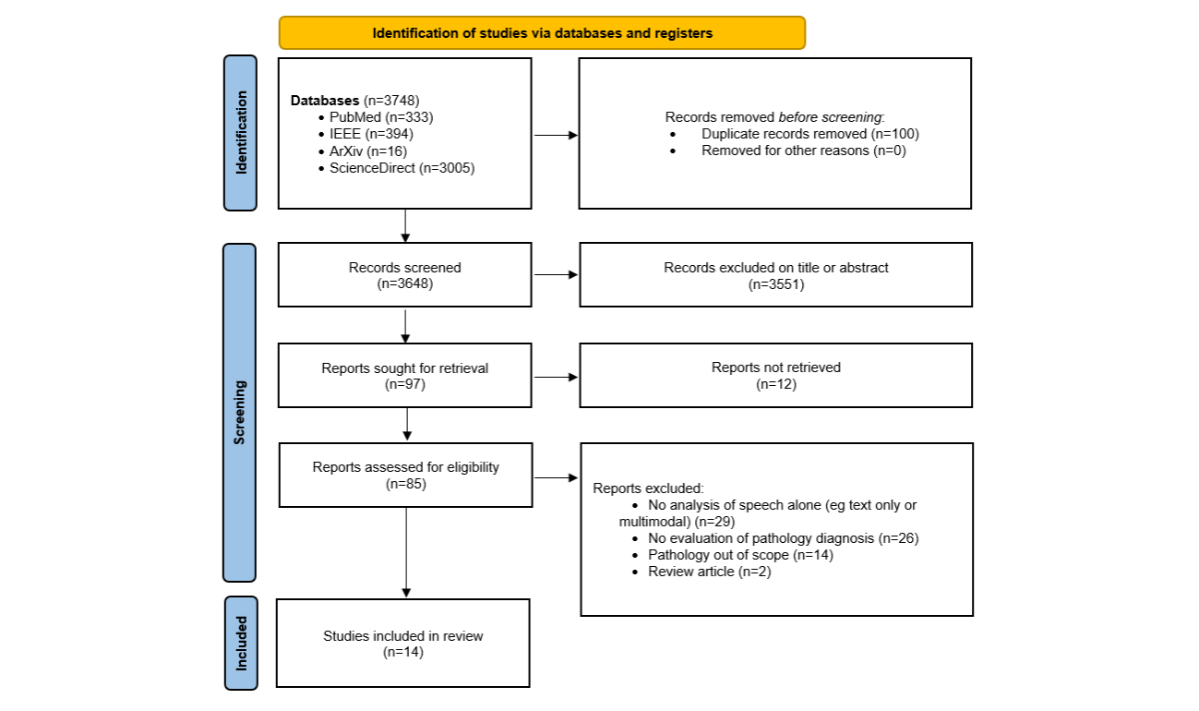
Flow diagram of the study selection process based on the PRISMA (Preferred Reporting Items for Systematic Reviews and Meta-Analyses) guidelines.

As outlined in the flowchart in Figure 3, a total of 3648 studies were screened, with 85 (2.33%) reports retrieved and assessed. From these 85 studies, 14 (20%) were included in the final review, with the most common reasons for exclusion being the lack of speech analysis alone (ie, models using only text or a combination of audio and other modalities) or the absence of a diagnostic perspective within the study (ie, no prediction of pathological severity or comparison between patients and controls). Of the 14 studies included in the final selection, 3 (18%) addressed suicide risk and suicidal ideation (SI), 8 (53%) analyzed depression and mood disorders, and 3 (18%) studied psychotic disorders.

Several studies screened did not analyze the emotional state of the patients but examined their capacity to detect emotional expressions in others, that is, to determine whether their pathologies impacted their perception of emotional expressions.

### Risk of Bias Assessment

**Figure 4:**
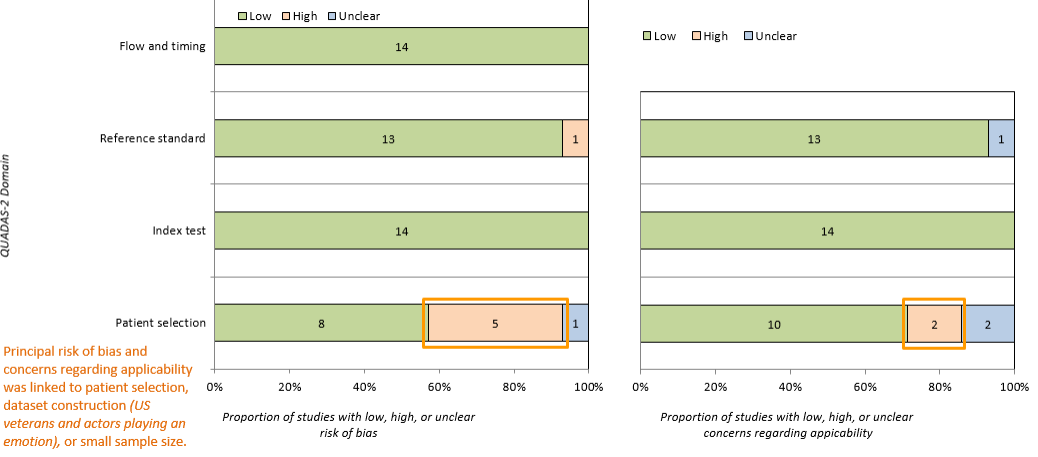
The Quality Assessment of Diagnostic Accuracy Studies 2 (QUADAS-2) results, highlighting risk of bias and applicability concerns. Patient selection was associated with a high risk of bias owing to sampling methods and sample size.

Most of the included studies had a low risk of bias across all fields. As shown in Figure 4, the main exception was in patient selection, where 5 studies were deemed to have a high risk of bias. Among these 5 studies, 3 selected participants only from within clinical populations (ie, no control group), and the remaining 2 did not select from a representative sample [3,18-21]. In addition, high concerns regarding the applicability of patient selection were noted in 2 cases [3,19]. 4 studies raised unclear concerns regarding patient selection [5,7,11]. The full Quality Assessment of Diagnostic Accuracy Studies–2 results are available in Multimedia Appendix 1.

This section is divided into 2 parts. First, we present a narrative review of established methodologies within the field of SER. Second, the results of the PRISMA review are presented.

### Narrative Review

Here, we present a brief overview of the methods and technical architectures used within the field of SER, covering the transition from traditional ML methods to NNs and state-of-the-art transformer-based methods [22].

### Established Methodologies

Speech, serving as a primary mode of communication, harbors a wealth of emotional cues that can be harnessed to assess an individual’s mental and emotional well-being. Various methodologies have been devised to extract, analyze, and interpret these cues, each providing distinct perspectives on the underlying emotional states.

#### Acoustic or Prosodic Feature Extraction

Acoustic or prosodic feature extraction involves the analysis of various acoustic properties of speech signals, such as pitch, intensity, duration, and spectral characteristics [1,2,23,24]. Acoustic features are related to the physical properties of sound waves and can be observed at the level of milliseconds, for example, frequency, which is related to how humans perceive pitch. Prosodic features are typically observed over a longer timescale, with pitch contours, for example, measuring how pitch changes over time, and other measures, including speech rate and pauses. Both sets of features serve as the basis for quantifying emotional cues present in speech and are commonly used as input for ML models. Techniques such as mel-frequency cepstral coefficients (MFCCs), pitch contour analysis, and formant analysis are commonly used in acoustic feature extraction for emotion detection tasks [25].

These measures correlate with various aspects of speech production, which can be impacted by the emotional state of the speaker. MFCCs, for example, capture the spectral characteristics of speech (ie, how energy is distributed across different frequencies), which can be linked to certain emotions (eg, higher energy in mid to high frequencies for anger or joy). Pitch contour analysis tracks variation in the fundamental frequency of speech, that is, the perceived pitch, over time. Variation in a speaker’s pitch allows listeners to distinguish between sadness and excitement. Finally, formant values are influenced by the shape and tension of the vocal tract during speech. Physiological aspects of emotions (eg, smiling) may impact these values.

#### The OpenSMILE Toolkit

Among the most prevalent tools used for acoustic feature extraction is the *openSMILE* toolkit [14]. This toolkit is available in both a Windows executable (.exe) file and a Python-accessible package and contains several widely used collections of acoustic and prosodic measures (referred to as feature sets). These feature sets encompass a variety of acoustic characteristics, such as frequency, loudness, MFCCs, and other speech attributes. These features can then be analyzed using the methods presented in subsequent sections to detect emotions or, in the context of computational paralinguistics, to aid in the recognition of mental health conditions. Among its strongest advantages are the ease of use for feature extraction and the standardization of feature sets, allowing for more straightforward comparison between results.

#### Traditional ML Approaches (Before NNs)

Traditional ML approaches involve the use of statistical and pattern recognition algorithms to classify emotional states based on extracted features. These approaches typically include algorithms such as support vector machines (SVM), k-nearest neighbor (KNN), and decision trees [26-30]. By training these models on labeled datasets of emotional speech samples, they can learn to classify new instances into predefined emotional categories.

#### Recurrent NNs: Long Short-Term Memory and Bidirectional Long Short-Term Memory

Recurrent NNs, particularly Long Short-Term Memory (LSTM) and bidirectional LSTM architectures, gained popularity in speech emotion detection tasks because of their ability to capture temporal dependencies in sequential data [31-34]. These NNs excel at processing time-series data, such as speech signals, allowing them to capture long-range dependencies and subtle temporal patterns in emotional expression.

#### Transformers

Transformers, originally developed for NLP tasks, have emerged as the state-of-the-art approach for analyzing speech signals for emotion detection [22,35-39]. These architectures, including models such as the Bidirectional Encoder Representations from Transformers, excel at capturing contextual information and semantic relationships within sequential data. By fine-tuning pretrained transformer models on emotion detection tasks, researchers can leverage their powerful language understanding capabilities to analyze emotional content in speech. One study involved the use of transformer, specifically, a self-attention-based deep learning (DL) model combining a 2-dimensional convolutional neural network and an LSTM network. This model focuses on optimizing feature extraction from speech using MFCCs and achieved an impressive average test accuracy of 90% [40].

### Available Datasets

A multitude of datasets for the SER task exist, each falling into one of several categories based on certain characteristics (eg, the type of data collected, the classification of emotions, or the relation to other tasks). However, few to no datasets are available that serve both SER and mental health applications. This section provides an overview of some of the most widely used datasets and their characteristics.

#### Distress Analysis Interview Corpus–Wizard of Oz

The Distress Analysis Interview Corpus–Wizard of Oz (DAIC-WOZ) dataset is part of the larger Distress Analysis Interview Corpus [41]. It includes audio and transcript recordings of semistructured clinical interviews between participants and an interviewer. The interviews were designed to detect signs of depression, anxiety, and posttraumatic stress disorder. Using scores from several questionnaires related to psychological distress and current mood, the audio and transcript data can be used for a classification of the interviewees.

While the dataset does not explicitly include annotations of the emotional states of the interviewees, labels could be derived using another SER model to directly analyze correlations between the participants’ emotional and mental states. A total of 193 interviews are available, with each interview lasting between 5 and 20 minutes, and all the interviews were conducted in North American English.

#### Ryerson Audio-Visual Database of Emotional Speech and Song

Ryerson Audio-Visual Database of Emotional Speech and Song is a multimodal database (including both audio and face recordings), containing both acted speech and song [42]. The recordings, in North American English, feature 24 professional actors (12 male and 12 female actors).

Each expression (ie, emotion) is produced at 2 levels of emotional intensity, as well as at a neutral level. The dataset comprises a total of 7365 recordings (4320 speech and 3036 singing).

#### FAU-AIBO Dataset

The FAU-AIBO dataset consists of spontaneous speech from German children’s interaction with a human-controlled robot, which they were told was autonomous [43].

The data is annotated on the word level for the presence of 11 emotional states, as evaluated by 5 judges.

This dataset was used in the initial 2009 INTERSPEECH SER Challenge [13]. Recent work has shown that this dataset presents a significant challenge in achieving strong classification performance, even for state-of-the-art methods [16].

#### Interactive Emotional Dyadic Motion Capture Dataset

The Interactive Emotional Dyadic Motion Capture dataset is a multimodal resource, including both audio and motion capture data, allowing analysis of both speech and facial expressions, and gestures [44]. The dataset comprises scripted speech as well as improvised scenarios, all produced by actors who were specifically instructed to produce or elicit certain emotions.

Both categorical and dimensional labeling approaches are used. The dataset contains approximately 12 hours of data.

#### Canadian French Emotional Dataset

The Canadian French Emotional dataset is a Canadian French emotional speech dataset [45]. It includes recordings from 6 male and 6 female actors reading 6 different sentences in the 6 basic emotional states described by Ekman and in a neutral state, containing approximately 69 minutes of recordings in total. While this dataset is smaller than many other available datasets, it highlights how few resources exist for languages other than English.

### PRISMA Selected Studies

The selected studies are presented in the subsequent sections, categorized based on mental health pathologies. Table S1 in Multimedia Appendix 2 provides a full overview of the included studies. This overview summarizes key characteristics such as methodology (eg, NNs, transformer models, or acoustic feature–based ML). Several trends can be observed, including the predominance of English and Mandarin datasets and the frequent use of LSTM and transformer models in recent years. The table also shows the potential risk of bias owing to population selection, as highlighted in the QUADAS assessment earlier. The details of each included study are presented in the subsequent sections.

#### Suicide Risk and SI

Gerczuk et al [19] explored differences in speech based on sex in the context of suicide risk, using both interpretable (ie, acoustic) and deep features. The authors reported the best results using an emotion fine-tuned wav2vec 2.0 model, achieving 81% balanced accuracy (ie, the model had an 81% chance of making a correct prediction for a given example, accounting for differences in sample sizes between groups) in high- versus low-risk suicide classification. Notably, this result was achieved by training the model separately for each sex. The authors reported a difference in the relationship between acoustic measures and suicide risk across sexes—agitation in male individuals was associated with increased suicide risk, whereas the opposite was true in female individuals—explaining the advantage of training the model separately for each group.

Another study analyzed interviews from patients who were recently discharged from the hospital after experiencing SI or other circumstances (ie, patients who had attempted suicide, those receiving psychiatric care, and healthy control participants) [20]. Two separate experiments were conducted. First, SER classifiers were trained and evaluated using acoustic features based on self-reported Positive and Negative Affect Schedule emotion labels from the interviews. Second, a comparison of the SI group and the other groups was performed based on the variability of the reported emotions.

The authors reported a maximum AUC of 0.78 when classifying the different emotion labels, and an AUC of 0.79 was achieved using the variability between these emotional states to distinguish between participants with SI and the other groups. The authors noted that the SI group showed lower emotional variability compared with the other groups, indicating that emotional states can be a good indicator of discrimination between control and pathological groups. These scores demonstrate a good level of discrimination between the groups, with AUC indicating the capacity of a model to differentiate between a randomly selected positive case and a randomly selected negative case (an AUC >0.7 indicates the model is achieving a fair discrimination rate, whereas an AUC of 0.5 is equivalent to random chance).

Suicidal ideas among US veterans were investigated by Belouali et al [3]. The study extracted a wide range of features (acoustic, prosodic, and linguistic) from recordings of veterans and trained several models (eg, random forest, logistic regression, and deep NNs). Feature selection was applied to identify the most relevant features for each model. The best results in classifying veterans with SI from their nonsuicidal counterparts were obtained using a combination of acoustic and linguistic features, achieving a sensitivity of 0.86, specificity of 0.70, and AUC of 0.80. The voices of individuals with SI differed from those of their nonsuicidal counterparts regarding energy (lower SD of energy contours in voiced segments, lower kurtosis, and lower skewness), indicating a flatter and less animated voice. Individuals with SI were also found to have more monotonous voices.

Overall, studies addressing suicide risk and SI demonstrated good discrimination between the patients with SI and control groups (AUC approximately 0.8 and accuracy approximately 80%), indicating that these methods can prove useful in a clinical context to identify patients at high risk of committing suicide.

#### Depression and Mood Disorders

Using both the emotional labels as well as the Patient Health Questionnaire-8 (PHQ-8) depression scores for each participant, Wang et al [6] attempted to find a link between speech features and depression scores as well as depression status (ie, participants with depression vs control). Several models were used: from traditional ML approaches (SVM and random forest) to transformer-based approaches (combining several complex models). The best performance was achieved by this complex transformer-based model, reaching an accuracy of 77% (ie, a 77% chance of assigning a given participant to the correct group) and an *F*_1_-score of 0.63, indicating the capability of complex models to outperform more traditional ML approaches on the same dataset.

Yang et al [46] highlighted how confusion between patients with low-mood bipolar depression and those with unipolar depression can lead to patients not receiving appropriate treatment. The patients were shown videos to elicit various emotions (happy, sad, disgust, fear, surprise, and anger) and asked to respond aloud to several questions. The recordings were labeled (with emotion profiles, ie, probabilities of each emotion for a given recording) using an SVM classifier trained on the eNTERFACE dataset. Different classifiers were then trained to distinguish between bipolar, unipolar, and healthy control groups based on these emotional profiles. The model (a combination of both LSTM and bidirectional LSTM architectures) achieved a classification accuracy of 77% when distinguishing among the 3 groups.

Correlations were found between vocal prosody measures and change in the measures of the Hamilton Rating Scale for Depression over the course of 21 weeks, as outlined in Yang et al [47]. The participants were diagnosed with major depressive disorder according to the *DSM-IV* guidelines, and the measures of switching pause (ie, the time between utterances from the patient and the interviewer) and fundamental frequency were taken from interviews conducted throughout the duration of the study. When analyzing within-subject variation in depression scores, the authors found that as depression severity decreased, pause duration became shorter and less variable, accounting for 32% of the overall variation over time in the patient’s depression scores. Furthermore, the depression scores obtained using hierarchical linear modeling, with linear discriminant classifiers reaching an accuracy of 69.5% when determining depression severity.

Stepanov et al [48] addressed the question of determining depression severity in the 2017 Audio-Visual Emotion Challenge using the DAIC-WOZ dataset. This challenge involved predicting PHQ-8 scores based on the patients’ speech. The best performance was achieved using low-level acoustic features extracted through openSMILE to train an LSTM model.

Extracting prosodic features (glottal flow, voice quality, and spectral features) from the DAIC-WOZ dataset, Mao et al [49] trained a range of DL models, with a hybrid model achieving an impressive accuracy of 98.7% and an *F*_1_-score of 0.987, indicating that the model could almost perfectly distinguish between the control and depression groups.

By applying the transformer architecture to frequency-related parameters from both the DAIC-WOZ dataset and their own proprietary data, Yang et al [50] achieved maximum *F*_1_-scores of 0.78 and 0.87, respectively. By analyzing the frequency components most important to their model’s predictions, the authors found that the frequency range from 600-700 Hz was the most important, corresponding to the Mandarin vowel /e/ or /ê/. The authors presented this as a potential biomarker for depression.

Studies applied to depression and mood disorders were the most prevalent among the results of this study (8 of the 14 selected articles). A range of different results were achieved, with newer methods showing strong improvement compared with older works (an accuracy of 69% in 2013 using only 2 prosodic features [47] vs 98% in 2023 using a range of prosodic features and DL methods [49]). These findings suggest that automated methods can be useful to clinicians for diagnostic purposes (notably for determining severity) and may help guide clinicians in adapting their therapeutic approaches.

#### Psychotic Disorders

Chakraborty et al [51] used the *emobase* feature set of low-level descriptors from openSMILE to discriminate between a group of patients with schizophrenia and a group of healthy controls, based on an interview conducted with a psychologist. In addition, a negative symptom assessment 16 (NSA-16) score, ranging from 1 to 6, was assigned by the psychologist to indicate the severity of negative symptoms of schizophrenia, that is, mainly motivational and emotional impairments. Prediction of these scores was also evaluated. A range of classifiers was trained on the data after conducting feature selection. The best-performing classifier for patient versus control classification was a linear SVM classifier using principal component analysis feature selection, reaching 79.49% accuracy (compared with 66.67% for a classifier predicting the majority class). For negative symptom assessment 16 prediction, classifiers were trained for each of the different items, with accuracy ranging from 62% to 85%. The best-performing classifiers were SVMs, KNN, and decision trees, with a range of different feature selection techniques.

Using a proprietary dataset of patients with schizophrenia with and without formal thought disorder (FTD), first-degree relatives, and neurotypical controls (15 of each; 60 total), Çokal et al [18] elicited spontaneous speech using an image description task. Pauses were measured in the participants’ speech and classified based on the duration of pause, the presence or absence of a filler word (eg, *umm* and *ehh*), and the syntactic context in which the pause occurred.

Patients without FTD produced significantly more unfilled pauses (ie, pauses without a filler word) than the controls in both utterance-initial contexts and before embedded clauses, and had more pauses before embedded clauses compared with patients with FTD. When compared with the control and first-degree relative groups, patients with FTD produced longer utterance-initial pauses.

The extended Geneva Minimalistic Acoustic Parameter Set (eGeMAPS) [52], available through openSMILE, was used by de Boer et al [53] along with a random forest classifier to distinguish between patients with schizophrenia spectrum disorders and healthy controls. The model achieved a classification accuracy of 86% between healthy controls and patients and an accuracy of 74% among patients between those with negative symptoms and those with positive symptoms. They also indicated that their work was a positive step toward validating language features as biomarkers in psychiatry.

Once again, the results from these studies applied in the context of psychosis performed well when distinguishing between groups, as well as among subgroups of patients with schizophrenia. In a clinical context, these methods can be of notable use for patient screening and early detection, areas in which clinicians often struggle to clearly orient their patients. It is worth noting that none of the works included in the reviewed sample involved a direct analysis of emotions in the context of psychotic disorders.

### Overview of Biomarkers in Mental Health Prediction

Several of the studies included in this review propose that the acoustic measures used could be considered as speech-derived biomarkers for mental health prediction. These biomarkers span the prosodic, spectral, and temporal domains and are often extracted using standardized tools, such as the openSMILE toolkit.

#### Prosodic and Temporal Markers

Markers, such as pitch (F0), energy, pause patterns, and speech rate, were commonly explored. For example, shorter and less variable within-subject pauses were associated with increasing depression severity [47]. Çokal et al [18] observed longer utterance-initial pauses in patients with FTD and more unfilled pauses in patients with schizophrenia without FTD. Belouali et al [3] reported lower energy variability and flatter energy contours in suicidal speech, suggesting a dull and monotonous prosody.

#### Spectral Features

Many studies relied on MFCCs, spectral slope, formants, and related features. Gerczuk et al [19] highlighted spectral slope (0-500 Hz), alpha ratio, and F1 bandwidth as predictive of suicide risk, with nuanced gender differences. Both F2 and spectral flux were found to be negatively associated with depression (indicating lower levels of energy and motivation), while a higher MFCC 4 was positively associated with depression [21]. Yang et al [50] emphasized specific frequency bands corresponding to Mandarin vowel formants that were positively associated with depression.

#### Feature Rankings and Model-Driven Importance

Some studies [19,53] analyzed feature relevance using importance rankings from models trained on the eGeMAPs and the ComParE feature set to identify features, such as voiced segments per second, spectral flux, and pitch percentiles, as top contributors for schizophrenia and depression, respectively. Stepanov et al [48] found spectral features to be more predictive than prosodic or voice quality features for PHQ score prediction.

While a few studies [46,51,54] applied acoustic features without further interpretation, this diversity of biomarker use demonstrates the strength of acoustic feature–based models as interpretable alternatives to end-to-end models.

## Discussion

### Principal Findings

Within the fields of psychology and psychiatry, the emotional states of patients can be linked to their condition. This is the case both in a pathological sense, with patients with depression being characterized as having low moods associated with sadness, or in a symptomatic sense, where the emotional state of a patient with depression can vary depending on their current circumstances. While several works exist at the intersection of these fields and the emerging field of SER, the diversity in methodologies, datasets, and results makes it difficult to determine what the practical application of these results in a clinical context could look like.

Nonetheless, the results presented in the selected studies are promising, with many of the models discussed earlier differentiating between pathological and healthy control groups at a significant rate (accuracy approximately 70%-80% and AUC approximately 0.8). The application of these technologies across a wide variety of domains is also promising, indicating that these technologies could be used for early screening (eg, in the diagnosis of psychotic disorders, where clinicians may struggle to guide patients early on).

The use of the *openSMILE* toolkit and its various feature sets (eg, eGeMAPS) throughout several of the selected studies highlights the possibility of integrating SER approaches within a clinical context. Furthermore, the results achieved using these feature sets are promising, especially given their ease of use and interpretation.

Recent research on the application of SER techniques in psychiatry appears promising, particularly in highlighting the potential clinical utility of such tools. As evidenced by several studies included in this review, SER can be used in between-subjects designs to differentiate patients from healthy controls or to distinguish between individuals with different psychiatric diagnoses, thereby providing support for the diagnostic process.

Beyond diagnostic discrimination, the potential of SER extends to within-subject applications, particularly in the context of longitudinal monitoring. For instance, SER systems could be used to assess the daily emotional states of individuals with various psychiatric conditions—such as major depressive disorder or borderline personality disorder—for example, integrated into ecological momentary assessment frameworks.

Moreover, SER technologies may hold predictive value with respect to clinical course, offering support in the monitoring of critical symptoms, such as SI, depressive, manic, or psychotic symptoms. This could help with the early identification and treatment of conditions that may pose significant risks to patients.

Finally, SER tools may serve as a complementary measure in the assessment of treatment efficacy, providing objective data on emotional expression that can enrich traditional clinical evaluations.

To the best of our knowledge, this study is the first systematic review to provide a synthesized overview of SER in psychiatry. This work could help standardize future studies and improve the reproducibility and comparability of results.

### Challenges and Limitations

While SER holds promise for health care applications, several challenges and limitations must be addressed to realize its full potential. One such challenge is the exploration of multimodal emotion recognition, which involves integrating multiple sources of emotional cues, including speech, body language, and facial expressions. While SER primarily focuses on analyzing speech signals, incorporating additional modalities could enhance the accuracy and robustness of emotion recognition systems, particularly in complex health care settings.

In addition, the interpretability of ML models and the ethical considerations surrounding the use of sensitive health data present significant challenges in the development and deployment of SER systems in health care settings. Ensuring transparency and accountability in the decision-making process of these algorithms is essential for building trust and confidence among health care professionals and patients. As outlined earlier, some complex models (ie, models whose added complexity makes clinical interpretation of predictions increasingly difficult) can achieve results superior to those of traditional ML methods applied to acoustic or prosodic features, which are more easily interpreted [6]. If these methods are to be applied within a clinical context, a trade-off must be found between classification performance and interpretability.

Furthermore, the generalization of SER algorithms across diverse populations and cultural contexts remains a significant hurdle. The major datasets used within the field of SER (eg, Interactive Emotional Dyadic Motion Capture) are in English, and as shown in Table S1 in Multimedia Appendix 2, most of the studies included in this review examined either English-speaking participants or those who spoke Mandarin or other Chinese languages. In all cases, these datasets are composed of participants from a single cultural context, which may limit their applicability to the expression of emotion across different cultures. Variations in speech patterns, dialects, and cultural norms can impact the performance of SER systems, highlighting the need for robust validation and adaptation strategies to ensure the reliability and effectiveness of these systems across different demographics [24].

In addition to these limitations, translating NLP results into routine clinical practice in psychiatry—implying rapid, replicable, and scalable analysis—presents specific challenges.

First, possible issues with the inaccuracy of vocal recording or transcription can hinder detailed analyses when integrating NLP tools into existing clinical workflows. Although linguistic analyses are generally considered fairly robust regarding the quality of the transcripts [55], conducting fine-grained analysis, such as SER, may require higher-quality data, which is not always available in a clinical context.

Addressing these challenges requires interdisciplinary collaboration among researchers, clinicians, and technologists to develop innovative solutions that prioritize patient privacy, data security, and ethical considerations while harnessing the potential of SER to enhance mental health assessment and treatment in health care settings.

### Future Directions

Separately, the tasks of SER and mental health screening have been addressed using methods that span statistical analysis of acoustic measures, ML, and DL approaches. These techniques have been applied to a range of pathologies, conditions, and disorders within the field of mental health. In this paper, we have specifically addressed those works that exclusively use the audio from the speaker’s speech as the input to their models. Some works have proposed that acoustic measures of patients’ speech be developed as biomarkers of pathology, as it is the clinician’s perception of these measures that allows a diagnosis [53]. This highlights these measures’ obvious advantage compared with the use of transformers and other DL models when collaborating in a clinical context: their ease of interpretation.

Among the various applications, similarities can be observed, notably in the reuse of certain methods, especially the use of acoustic measures, both in mental health and SER, which can be linked back to the emotional state of the patient. Given that the same methods are used both for the recognition of pathologies and the recognition of participants’ emotional states, the question arises: Could the direct automated analysis of patients’ emotional states be useful for investigating the aforementioned pathologies?

#### Proposed Pipelines for Audio Processing

Here, two possible approaches are proposed for the detection of pathologies from patients’ speech. From our review, most studies adopt the first approach described in the subsequent section, which involves mapping directly from a patient’s speech to a possible pathology.

##### Speech to Pathology

This pipeline is represented in the vast majority of studies in the literature, in which feature extraction or ML and DL methods are applied directly to the signal from recordings of the participants.

##### Speech to Emotion and Emotion to Pathology

In this approach, speech from a patient is first analyzed using a SER system (based on one of the various methodologies outlined earlier). The emotional states recognized by this system are then analyzed to gain insight into the patient’s pathological status. Although some studies have adopted a similar approach [20], this approach for the detection of mental health conditions has not been widely explored.

The pipeline from speech to emotion, then emotion to pathology, presents a notable advantage in a clinical context, because it offers a clear interpretation of why a given classification was made. Furthermore, this approach can be used collaboratively with health care professionals to facilitate and complement their work.

One possible challenge associated with this approach is the introduction of error by the SER system. To mitigate this risk, the selection of the SER system is of utmost importance, ensuring that its design (eg, training data) allows it to perform effectively on a given set of data.

##### Shared Challenges

From our review, the variety of methods observed (eg, a wide range of audio processing methods and feature sets, datasets of different sizes and compositions, and the use of various feature sets) led to difficulties in directly comparing different studies, even those applied to the same pathologies. This difficulty in comparison is similar to that encountered during the early days of the field of SER.

Further emphasis on the organization of shared challenges could promote the sharing of methods, data, and results, thus, improving the comparability of work in the field, as was the case in SER. Several challenges have been conducted within this field (or adjacent ones), with the most recent including the Audio-Visual Emotion Challenge 2019 for depression [56] and the Alzheimer dementia recognition through spontaneous speech challenge for Alzheimer disease [57].

One hurdle to overcome in the organization of these challenges, especially in a context relating to health care, is the sharing of confidential data.

#### Dimensional Approach to Pathology

Dimensional approaches to pathology in mental health can offer a more refined perspective on disorders by focusing on symptom severity and underlying mechanisms rather than rigid diagnostic categories.

To be consistent with the papers we reported, we grouped mental health conditions in this review according to disease categories. In contrast to this categorical approach, a dimensional approach could provide different insights [58]. In practice, this paradigm shift is conducive to the identification of (linguistic) biomarkers [59]. This conceptual change in understanding psychiatric disorders aims to facilitate collaborative work by proposing a common framework through which specialists from different fields can study pathological mechanisms.

Thus, the RDoC classification in psychiatry, described previously, proposes a dimensional understanding of mental disorders, facilitating the understanding of individual variability and accounting for the different clinical presentations among patients with the same pathology from a categorical perspective.

In the same way, the Hierarchical Taxonomy of Psychopathology (HiTOP), proposed by Kotov et al [60], is a promising model, especially for SER. Rather than replacing traditional categorical classifications, HiTOP hierarchically organizes symptoms into spectra and subfactors. It conceptualizes mental disorders along broad dimensions that reflect underlying emotional and behavioral patterns.

Internalized disorders are characterized by self-directed emotional and psychological symptoms, such as anxiety, depression, or phobias. Individuals affected by these disorders tend to internalize their struggles, often isolating their emotions. Externalized disorders, on the other hand, manifest in outwardly directed disruptive behaviors, such as aggression, impulsivity, or transgression of social norms. Thought disorders (eg, schizophrenia) are characterized by confusion, detachment from reality, and unusual experiences. By framing psychopathology as a continuum, HiTOP moves beyond discrete diagnostic categories to capture the overlapping nature of emotional and behavioral dysfunctions.

At the same time, the integration of NLP and emotion recognition into this approach is opening new perspectives. NLP enables the analysis of language data by detecting linguistic cues associated with mental disorders, such as depression or anxiety. These analyses facilitate the extraction of psychological and emotional dimensions, enriching the RDoC criteria with behavioral and cognitive data. The use of linguistic data in the RDoC approach could thus improve diagnostic processes, offering a more detailed and dynamic view of psychiatric disorders, and contribute to more personalized treatments adapted to the specific profiles of each patient. This convergence of neuroscientific and technological advances could truly transform the way psychiatry approaches mental disorders.

It should be stressed that proposing a clear and systematic mapping from emotions to dimensional approaches to pathologies remains a complex task. Emotional models in SER are based on theoretical presuppositions derived from psychology or cognitive science, while psychiatric classification systems rely on distinct clinical and diagnostic logics, with sometimes divergent levels of granularity and objectives. For example, the circumplex model offers a fine-grained, continuous representation of emotions along the valence and arousal axes, but does not directly align with the diagnostic criteria of the *DSM* or the functional domains of the RDoC.

#### Multimodal Approaches

This study was limited to works that involved the analysis of audio exclusively; however, some of the included studies showed that a multimodal approach could outperform approaches based solely on the speech signal (eg, Mao et al [49]). Many architectures have been proposed; one such example is the contrastive language-audio pretraining architecture [61]. This model is based on the transformer architecture and is trained on audio and text pairs using 2 encoders. In its introduction, this model was shown to outperform state-of-the-art models in several speech-related tasks without the need to train the model on data related to those tasks (a technique referred to as 0-shot learning).

While these approaches can be more effective than audio-only methods, it is worth noting that they can introduce more invasiveness to procedures, for example, through the recording of video.

### Conclusions

SER is emerging as a promising tool in mental health care, offering potential for early detection, continuous monitoring, and personalized interventions. This approach is based on ML and artificial intelligence technologies and involves the recognition of emotions from recordings of patients in a health care context, followed by the analysis of these emotions, with possible applications of other ML methods to either the emotions from the model or the use of the output of the SER model as input into a classifier to distinguish between populations (eg, control or pathological groups, or other classes fitting the dataset at hand).

This is the approach we suggest in the framework of the Apprentissage Profond pour l’Analyse Informatisée de la Subjectivité et des Emotions dans les troubles psychotiques émergents (DL for digital analysis of subjectivity and emotions in emerging psychotic disorders) project. This project, a collaboration between Sorbonne University, the French National Institute of Health, and the Brest University Hospital Centre, aims to develop automated methods for predicting psychotic transition among patients and, in doing so, improve the prognosis for patients. With this program, we aim to better understand the convergences and discontinuities between dimensional approaches, such as the RDoC, and models of emotion, to contribute to the development of speech analysis tools that are more aligned with clinical needs.

SER presents several advantages in this context, as an objective, noninvasive technique that potentially offers real-time insights into patients’ emotional states, with a promising role in diagnosis support and clinical evolution monitoring.

However, to be successful, interdisciplinary collaboration among computer scientists, psychologists, linguists, and health care professionals will be essential to refine these technologies and ensure their precise, nonbiased, and ethical implementation. Further work should explore the within-subject application of SER, particularly in the context of longitudinal monitoring. This approach would allow the regular assessment of patients’ emotional states and thus early identification and treatment of conditions, such as SI, depression, or psychotic episodes.

Beyond that, the next challenge for SER applications in mental health will likely be to integrate SER with other clinical data, as well as biological, genetic, and imaging data, in large-scale multimodal analyses to better characterize and predict psychiatric disorders.

## Appendix

Multimedia Appendix 1 The full Quality Assessment of Diagnostic Accuracy Studies 2 results.

Multimedia Appendix 2 PRISMA selected studies overview table.

Multimedia Appendix 3 PRISMA 2020 checklist.

## Abbreviations

AUC: area under the curve
DAIC-WOZ: Distress Analysis Interview Corpus–Wizard of Oz
DL: deep learning
DSM: Diagnostic and Statistical Manual of Mental Disorders
eGeMAPs: extended Geneva Minimalistic Acoustic Parameter Set
FTD: formal thought disorder
HiTOP: Hierarchical Taxonomy of Psychopathology
LSTM: Long Short-Term Memory
MFCC: Mel-frequency cepstral coefficient
ML: machine learning
NLP: natural language processing
NN: neural network
openSMILE: open-source Speech and Music Interpretation by Large-Space Extraction
PHQ-8: Patient Health Questionnaire-8
PRISMA: Preferred Reporting Items for Systematic Reviews and Meta-Analyses
RDoC: research domain criteria
SER: speech emotion recognition
SI: suicidal ideation
SVM: support vector machine

## References

[1] Latif S, Qadir J, Qayyum A, Usama M, Younis S (2021). Speech technology for healthcare: opportunities, challenges, and state of the art. IEEE Rev Biomed Eng.

[2] Ozseven T, Arpacioglu M (2024). Comparative performance analysis of metaheuristic feature selection methods for speech emotion recognition. Meas Sci Rev.

[3] Belouali A, Gupta S, Sourirajan V, Yu J, Allen N, Alaoui A, Dutton MA, Reinhard MJ (2021). Acoustic and language analysis of speech for suicidal ideation among US veterans. BioData Min.

[4] Huang KY, Wu CH, Su MH, Chou CH (2017). Mood disorder identification using deep bottleneck features of elicited speech. Proceedings of the Asia-Pacific Signal and Information Processing Association Annual Summit and Conference.

[5] Milling M, Baird A, Bartl-Pokorny KD, Liu S, Alcorn AM, Shen J, Tavassoli T, Ainger E, Pellicano E, Pantic M, Cummins N, Schuller BW (2022). Evaluating the impact of voice activity detection on speech emotion recognition for autistic children. Front Comput Sci.

[6] Wang H, Liu Y, Zhen X, Tu X (2021). Depression speech recognition with a three-dimensional convolutional network. Front Hum Neurosci.

[7] Dellaert F, Polzin T, Waibel A (1996). Recognizing emotion in speech. Proceeding of Fourth International Conference on Spoken Language Processing.

[8] Schuller BW (2018). Speech emotion recognition: two decades in a nutshell, benchmarks, and ongoing trends. Commun ACM.

[9] Ekman P, Sorenson ER, Friesen WV (1969). Pan-cultural elements in facial displays of emotion. Science.

[10] Plutchik R (1980). Emotion: A Psychoevolutionary Synthesis.

[11] Russell JA (1980). A circumplex model of affect. J Pers Soc Psychol.

[12] Plaza-del-Arco FM, Cercas Curry AA, Cercas Curry A, Hovy D (2024). Emotion analysis in NLP: trends, gaps and roadmap for future directions. Proceedings of the 2024 Joint International Conference on Computational Linguistics, Language Resources and Evaluation.

[13] Schuller B, Steidl S, Batliner A (2009). The INTERSPEECH 2009 emotion challenge. Proceedings of the 10th Annual Conference of the International Speech Communication Association.

[14] Eyben F, Wöllmer M, Schuller B (2010). Opensmile: the Munich versatile and fast open-source audio feature extractor. Proceedings of the 18th ACM International Conference on Multimedia.

[15] Abdel-Hamid O, Mohamed AR, Jiang H, Penn G (2012). Applying Convolutional Neural Networks concepts to hybrid NN-HMM model for speech recognition. Proceedings of the IEEE International Conference on Acoustics, Speech and Signal Processing.

[16] Triantafyllopoulos A, Batliner A, Rampp S, Milling M, Schuller B (2025). INTERSPEECH 2009 emotion challenge revisited: benchmarking 15 years of progress in speech emotion recognition. ArXiv. Preprint posted online on June 10, 2024.

[17] Cuthbert BN, Insel TR (2013). Toward the future of psychiatric diagnosis: the seven pillars of RDoC. BMC Med.

[18] Çokal D, Zimmerer V, Turkington D, Ferrier N, Varley R, Watson S, Hinzen W (2019). Disturbing the rhythm of thought: speech pausing patterns in schizophrenia, with and without formal thought disorder. PLoS One.

[19] Gerczuk M, Amiriparian S, Lutz J, Strube W, Papazova I, Hasan A, Schuller BW (2025). Exploring gender-specific speech patterns in automatic suicide risk assessment. ArXiv. Preprint posted online on June 26, 2024.

[20] Gideon J, Schatten HT, McInnis MG, Provost EM (2019). Emotion recognition from natural phone conversations in individuals with and without recent suicidal ideation. Proceedings of the INTERSPEECH 2019.

[21] Zhou Y, Han W, Yao X, Xue J, Li Z, Li Y (2023). Developing a machine learning model for detecting depression, anxiety, and apathy in older adults with mild cognitive impairment using speech and facial expressions: a cross-sectional observational study. Int J Nurs Stud.

[22] Khare SK, Blanes-Vidal V, Nadimi ES, Acharya UR (2024). Emotion recognition and artificial intelligence: a systematic review (2014–2023) and research recommendations. Inf Fusion.

[23] Zhao S, Yang Y, Cohen I, Zhang L (2021). Speech emotion recognition using auditory spectrogram and cepstral features. Proceedings of the 29th European Signal Processing Conference.

[24] de Lope J, Graña M (2023). An ongoing review of speech emotion recognition. Neurocomputing.

[25] Liu GK (2025). Evaluating gammatone frequency cepstral coefficients with neural networks for emotion recognition from speech. ArXiv. Preprint posted online on June 23, 2018.

[26] Al Dujaili MJ, Ebrahimi-Moghadam A, Fatlawi A (2021). Speech emotion recognition based on SVM and KNN classifications fusion. Int J Electr Comput Eng.

[27] Akinpelu S, Viriri S (2023). Speech emotion classification using attention based network and regularized feature selection. Sci Rep.

[28] Ismaiel W, Alhalangy A, Mohamed AO, Musa AI (2024). Deep learning, ensemble and supervised machine learning for Arabic speech emotion recognition. Eng Technol Appl Sci Res.

[29] Al Dujaili MJ, Ebrahimi-Moghadam A (2023). Automatic speech emotion recognition based on hybrid features with ANN, LDA and K_NN classifiers. Multimed Tools Appl.

[30] Nath S, Shahi AK, Martin T, Choudhury N, Mandal R (2024). Speech emotion recognition using machine learning: a comparative analysis. SN Comput Sci.

[31] Abdelhamid AA, El-Kenawy EM, Alotaibi B, Amer GM, Abdelkader MY, Ibrahim A, Eid MM (2022). Robust speech emotion recognition using CNN+LSTM based on stochastic fractal search optimization algorithm. IEEE Access.

[32] Soltau H, Liao H, Sak H (2025). Neural speech recognizer: acoustic-to-word LSTM model for large vocabulary speech recognition. ArXiv. Preprint posted online on October 31, 2016.

[33] Senthilkumar N, Karpakam S, Gayathri Devi M, Balakumaresan R, Dhilipkumar P (2022). Speech emotion recognition based on bi-directional LSTM architecture and deep belief networks. Mater Today Proc.

[34] Etienne C, Fidanza G, Petrovskii A, Devillers L, Schmauch B (2025). CNN+LSTM architecture for speech emotion recognition with data augmentation. ArXiv. Preprint posted online on February 15, 2018.

[35] Lee S, Han DK, Ko H (2020). Fusion-ConvBERT: parallel convolution and BERT fusion for speech emotion recognition. Sensors (Basel).

[36] Gong Y, Chung YA, Glass J (2025). AST: audio spectrogram transformer. ArXiv. Preprint posted online on April 5, 2021.

[37] Zhang Z, Wu B, Schuller B (2025). Attention-augmented end-to-end multi-task learning for emotion prediction from speech. ArXiv. Preprint posted online on March 29, 2019.

[38] Ullah R, Asif M, Shah WA, Anjam F, Ullah I, Khurshaid T, Wuttisittikulkij L, Shah S, Ali SM, Alibakhshikenari M (2023). Speech emotion recognition using convolution neural networks and multi-head convolutional transformer. Sensors (Basel).

[39] Yoon S, Byun S, Jung K (2025). Multimodal speech emotion recognition using audio and text. ArXiv. Preprint posted online on October 10, 2018.

[40] Singh J, Saheer LB, Faust O (2023). Speech emotion recognition using attention model. Int J Environ Res Public Health.

[41] Gratch J, Artstein R, Lucas G, Stratou G, Scherer S, Nazarian A, Wood R, Boberg J, DeVault D, Marsella S, Traum D, Rizzo S, Morency LP (2014). The distress analysis interview corpus of human and computer interviews. Proceedings of the Ninth International Conference on Language Resources and Evaluation.

[42] Livingstone SR, Russo FA (2018). The Ryerson Audio-Visual Database of Emotional Speech and Song (RAVDESS): a dynamic, multimodal set of facial and vocal expressions in North American English. PLoS One.

[43] Steidl S (2009). Automatic Classification of Emotion-related User States in Spontaneous Children's Speech.

[44] Busso C, Bulut M, Lee CC, Kazemzadeh A, Mower E, Kim S, Chang JN, Lee S, Narayanan SS (2008). IEMOCAP: interactive emotional dyadic motion capture database. Lang Resour Eval.

[45] Gournay P, Lahaie O, Lefebvre R (2018). A Canadian French emotional speech dataset. Proceedings of the 9th ACM Multimedia Systems Conference.

[46] Yang TH, Wu CH, Huang KY, Su MH (2016). Detection of mood disorder using speech emotion profiles and LSTM. Proceedings of the 10th International Symposium on Chinese Spoken Language Processing.

[47] Yang Y, Fairbairn C, Cohn JF (2013). Detecting depression severity from vocal prosody. IEEE Trans Affect Comput.

[48] Stepanov E, Lathuiliere S, Chowdhury SA, Ghosh A, Vieriu RL, Sebe N, Riccardi G (2025). Depression severity estimation from multiple modalities. ArXiv. Preprint posted online on November 10, 2017.

[49] Mao K, Zhang W, Wang DB, Li A, Jiao R, Zhu Y, Wu B, Zheng T, Qian L, Lyu W, Ye M, Chen J (2023). Prediction of depression severity based on the prosodic and semantic features with bidirectional LSTM and time distributed CNN. IEEE Trans Affect Comput.

[50] Yang W, Liu J, Cao P, Zhu R, Wang Y, Liu JK, Wang F, Zhang X (2023). Attention guided learnable time-domain filterbanks for speech depression detection. Neural Netw.

[51] Chakraborty D, Yang Z, Tahir Y, Maszczyk T, Dauwels J, Thalmann N, Zheng J, Maniam Y, Amirah N, Tan BL, Lee J (2018). Prediction of negative symptoms of schizophrenia from emotion related low-level speech signals. Proceedings of the IEEE International Conference on Acoustics, Speech and Signal Processing.

[52] Eyben F, Scherer KR, Schuller BW, Sundberg J, Andre E, Busso C, Devillers LY, Epps J, Laukka P, Narayanan SS, Truong KP (2016). The Geneva Minimalistic Acoustic Parameter Set (GeMAPS) for voice research and affective computing. IEEE Trans Affect Comput.

[53] de Boer JN, Voppel AE, Brederoo SG, Schnack HG, Truong KP, Wijnen FN, Sommer IE (2021). Acoustic speech markers for schizophrenia-spectrum disorders: a diagnostic and symptom-recognition tool. Psychol Med.

[54] Hansen L, Zhang YP, Wolf D, Sechidis K, Ladegaard N, Fusaroli R (2022). A generalizable speech emotion recognition model reveals depression and remission. Acta Psychiatr Scand.

[55] Corcoran CM, Mittal VA, Bearden CE, E Gur R, Hitczenko K, Bilgrami Z, Savic A, Cecchi GA, Wolff P (2020). Language as a biomarker for psychosis: a natural language processing approach. Schizophr Res.

[56] Ringeval F, Schuller B, Valstar M, Cummins N, Cowie R, Tavabi L, Schmitt M, Alisamir S, Amiriparian S, Messner EM, Song S, Liu S, Zhao Z, Mallol-Ragolta A, Ren Z, Soleymani M, Pantic M (2019). AVEC 2019 workshop and challenge: state-of-mind, detecting depression with AI, and cross-cultural affect recognition. Proceedings of the 9th International on Audio/Visual Emotion Challenge and Workshop.

[57] Luz S, Haider F, de la Fuente S, Fromm D, MacWhinney B (2025). Alzheimer's dementia recognition through spontaneous speech: the ADReSS challenge. ArXiv. Preprint posted online on April 14, 2020.

[58] Insel T, Cuthbert B, Garvey M, Heinssen R, Pine DS, Quinn K, Sanislow C, Wang P (2010). Research domain criteria (RDoC): toward a new classification framework for research on mental disorders. Am J Psychiatry.

[59] Kelly JR, Clarke G, Cryan JF, Dinan TG (2018). Dimensional thinking in psychiatry in the era of the Research Domain Criteria (RDoC). Ir J Psychol Med.

[60] Kotov R, Krueger RF, Watson D, Achenbach TM, Althoff RR, Bagby RM, Brown TA, Carpenter WT, Caspi A, Clark LA, Eaton NR, Forbes MK, Forbush KT, Goldberg D, Hasin D, Hyman SE, Ivanova MY, Lynam DR, Markon K, Miller JD, Moffitt TE, Morey LC, Mullins-Sweatt SN, Ormel J, Patrick CJ, Regier DA, Rescorla L, Ruggero CJ, Samuel DB, Sellbom M, Simms LJ, Skodol AE, Slade T, South SC, Tackett JL, Waldman ID, Waszczuk MA, Widiger TA, Wright AG, Zimmerman M (2017). The Hierarchical Taxonomy of Psychopathology (HiTOP): a dimensional alternative to traditional nosologies. J Abnorm Psychol.

[61] Elizalde B, Deshmukh S, Ismail MA, Wang H (2025). CLAP: learning audio concepts from natural language supervision. ArXiv. Preprint posted online on June 9, 2022.

