# Supplementary material for: Speech Emotion Recognition in Mental Health: Systematic Review of Voice-Based Applications
Source: JMIR Ment Health. 2025 Sep 30;12:e74260. doi: 10.2196/74260 (PMC12521853; doi:10.2196/74260)
Supplement: Multimedia Appendix 2 [file mental_v12i1e74260_app2.pdf]

Appendices

Study	*Methodology*	*Language*	*Population*	*Data Type*	*Disorder Studied*	*Sample Size*	*Principal findings*
**Belouali et al.** *[1]*	Acoustic + Linguistic Features, Deep Learning	English	US Veterans	Longitudinal audio diaries via mobile app	Suicide, PTSD	124 veterans, 588 recordings	Acoustic: AUC 0.78, Linguistic: AUC 0.74, Combined: AUC 0.80, Sensitivity 86%, Specificity 70%.
**Cokal et al.** *[2]*	Psycholinguistic Analysis (Speech Pausing)	English	UK patients (SZ+FTD, SZ-FTD), FDR, NC	Spontaneous speech (comic strip narration)	Schizophrenia (with and without FTD)	60 total	Significant differences in speech pause patterns ($p < 0.05$); SZ-FTD: more pauses >1s ($p=0.004$), SZ+FTD: more pauses before embedded clauses ($p<0.01$). No AUC/sens/spec reported.
**De Boer et al.** *[3]*	OpenSMILE Acoustic Features + Random Forest	English	Patients with schizophrenia-spectrum disorders	PANSS-based interviews	Schizophrenia-spectrum disorders	142 patients, 142 controls	Diagnosis: Accuracy 86.2%, Positive vs Negative symptoms classification accuracy: 74.2%.

Study	*Methodology*	*Language*	*Population*	*Data Type*	*Disorder Studied*	*Sample Size*	*Principal findings*
Gerczuk et al. *[4]*	wav2vec2.0, Gender-based Modelling, OpenSMILE	German	Suicidal patients, general population	Neutral text readings	Suicide Risk	20 patients (10 men and 10 women)	Gender-based model for suicidal risk classification: Accuracy 81%; High suicidality in men positively correlates with paralinguistic characteristics that are usually associated with a high arousal, agitated affective state while the speech patterns of female subjects point in the opposite direction.
Gideon et al. *[5]*	Acoustic Features, Neural Networks	English	Psychiatric hospital discharged individuals	Natural phone call recordings via smartphone app (EMASS)	Suicidal Ideation	43 individuals, 4078 calls	AUC =0.79 for suicidal ideation detection
Hansen et al. *[6]*	Transfer Learning, Mixture of Experts, Speech Emotion Recognition (SER)	Danish		*Training dataset:* public CREMA-D, RAVDESS, and EMO-DB<br><br>*Test evaluation dataset:* Clinical interview speech	Major Depressive Disorder (MDD)	42 controls, 40 MDD, 25 remission	AUC 0.71 for MDD vs. Controls, No significant difference between remission and controls.
Mao et al. *[7]*	Bi-LSTM, Time-Distributed CNN, Attention Mechanisms	English	US citizens and veterans suffering from depression, PTSD and anxiety	DAIC-WOZ dataset	Depression Severity	189 participants	Bi-LSTM: F1 =0.9870 (audio), F1 =0.9709 (text); Multimodal fusion: F1 =0.9580

Study	Methodology	Language	Population	Data Type	Disorder Studied	Sample Size	Principal findings
**Yang et al.** *[8]*	Attention-guided learnable time-domain filterbanks (DALF)	Mandarin		*Training dataset* Neutral Reading-based Audio Corpus (NRAC), private. *Test-evaluation dataset :* DAIC-WOZ and NRAC	Depression detection, no control group	NRAC dataset and DAIC-WOZ, sample sizes not specified	F1 scores for DALF model: 78.4% on DAIC-WOZ then 87.3% and 81.7% on NRAC. Most important frequency range identified by their method is 600–700Hz
**Wang et al.** *[9]*	3D Convolutional Network, Bi-GRU with Attention	Chinese	Depressed patients from Beijing hospitals, DAIC-WOZ participants	*Training dataset:* DAIC-WOZ *Test-evaluation dataset:* Cropped DAIC-WOZ as follow DAIC-ori (original data) and DAIC-mute-removed (data without mute segments)	Depression	76 patients, 189 interactions	3D-CBHGA model accuracy 74.29%
**Zhou et al.** *[10]*	Random Forest, Multiclass Classification	Mandarin		Private dataset from memory outpatient department	Depression, anxiety, apathy in MCI patients, no control group	319 older adults with mild cognitive impairment	Classification model F1 score: 96.6%, Accuracy: 87.4%, Precision: 86.6%, Recall: 87.6% for depression, anxiety, apathy classification

Study	*Methodology*	*Language*	*Population*	*Data Type*	*Disorder Studied*	*Sample Size*	*Principal findings*
Yang et al. *[11]*	Hierarchical Linear Modeling (HLM), Vocal Prosody Analysis	English	Recruitment from a depression trial	Semi-structured clinical interviews	Major Depressive Disorder (MDD)	49 individuals	Prosody features explained ~60% of depression score variance; severity classification accuracy 69% (κ=0.53).
Chakraborty et al. *[12]*	OpenSMILE Feature Extraction, Supervised Learning	English	Patients from Institute of Mental health in Singapore	Semi-structured clinical interviews	Schizophrenia (Negative Symptoms)	78 participants, 52 schizophrenia patients, 26 controls	Patient vs Control classification accuracy ranged between 60-85%.
Stepanov et al. *[13]*	OpenSMILE, LSTM<br>Multimodal (Speech, Language, Facial Features)	English	DAIC-WOZ participants	Multimodal (Speech, Language, Facial Features)	Depression Severity	Recordings from 189 sessions of interaction with a virtual psychologist	Spectral features were the best suited for classifying PHQ-8 scores in the development set : MAE= 4.96, RMSE= 6.32
Yang et al. *[14]*	LSTM, Emotion Profile Generation, SVM	Mandarin	CHI-MEI Mood Database: 39 subjects (13 BD, 13 UD, 13 healthy)	*Training dataset:* eNTERFACE database *Test-evaluation dataset :* CHI-MEI Mood Database	Mood Disorders Bipolar, Unipolar Depression	1170 responses from 39 subjects	LSTM: 0.78 accuracy for mood disorder classification

Table S1. Synthesis of results; SER (speech emotion recognition), CHR (clinical high risk), FTD (Formal Thought Disorder) CNN (Convolutional Neural Network) , MFCC, SVM (Support Vector Machine), LSTM-based VAD (Long Short-Term Memory-based Voice Activity Detection) , NLP (Natural Language Processing), DNN (Deep Neural Networks), POS Tagging (Part-Of-Speech Tagging), CCC (concordance correlation coefficient), RMSE (root mean square error)

This is a Multimedia Appendix to a full manuscript published in the J Med Internet Res. For full copyright and citation information see http://dx.doi.org/10.2196/jmir.xxxx

References

1. Belouali A, Gupta S, Sourirajan V, Yu J, Allen N, Alaoui A, Dutton MA, Reinhard MJ. Acoustic and language analysis of speech for suicidal ideation among US veterans. BioData Min 2021 Feb 2;14(1):11. doi: 10.1186/s13040-021-00245-y

2. Çokal D, Zimmerer V, Turkington D, Ferrier N, Varley R, Watson S, Hinzen W. Disturbing the rhythm of thought: Speech pausing patterns in schizophrenia, with and without formal thought disorder. Chialvo DR, editor. PLOS ONE 2019 May 31;14(5):e0217404. doi: 10.1371/journal.pone.0217404

3. De Boer JN, Voppel AE, Brederoo SG, Schnack HG, Truong KP, Wijnen FNK, Sommer IEC. Acoustic speech markers for schizophrenia-spectrum disorders: a diagnostic and symptom-recognition tool. Psychol Med 2023 Mar;53(4):1302–1312. doi: 10.1017/S0033291721002804

4. Gerczuk M, Amiriparian S, Lutz J, Strube W, Papazova I, Hasan A, Schuller BW. Exploring Gender-Specific Speech Patterns in Automatic Suicide Risk Assessment. Interspeech 2024 ISCA; 2024. p. 1095–1099. doi: 10.21437/Interspeech.2024-1097

5. Gideon J, Schatten HT, McInnis MG, Provost EM. Emotion Recognition from Natural Phone Conversations in Individuals with and without Recent Suicidal Ideation. Interspeech 2019 ISCA; 2019. p. 3282–3286. doi: 10.21437/Interspeech.2019-1830

6. Hansen L, Zhang Y, Wolf D, Sechidis K, Ladegaard N, Fusaroli R. A generalizable speech emotion recognition model reveals depression and remission. Acta Psychiatr Scand 2022 Feb;145(2):186–199. doi: 10.1111/acps.13388

7. Mao K, Zhang W, Wang DB, Li A, Jiao R, Zhu Y, Wu B, Zheng T, Qian L, Lyu W, Ye M, Chen J. Prediction of Depression Severity Based on the Prosodic and Semantic Features with Bidirectional LSTM and Time Distributed CNN. IEEE Trans Affect Comput 2023 Jul 1;14(3):2251–2265. doi: 10.1109/TAFFC.2022.3154332

8. Yang W, Liu J, Cao P, Zhu R, Wang Y, Liu JK, Wang F, Zhang X. Attention guided learnable time-domain filterbanks for speech depression detection. Neural Netw 2023 Aug;165:135–149. doi: 10.1016/j.neunet.2023.05.041

9. Wang H, Liu Y, Zhen X, Tu X. Depression Speech Recognition With a Three-Dimensional Convolutional Network. Front Hum Neurosci 2021 Sep 30;15:713823. doi: 10.3389/fnhum.2021.713823

10. Zhou Y, Han W, Yao X, Xue J, Li Z, Li Y. Developing a machine learning model for detecting depression, anxiety, and apathy in older adults with mild cognitive impairment using speech and facial expressions: A cross-sectional observational study. Int J Nurs Stud 2023 Oct;146:104562. doi: 10.1016/j.ijnurstu.2023.104562

11. Yang Y, Fairbairn C, Cohn JF. Detecting Depression Severity from Vocal Prosody. IEEE Trans Affect Comput 2013 Apr;4(2):142–150. doi: 10.1109/T-AFFC.2012.38

12. Chakraborty D, Yang Z, Tahir Y, Maszczyk T, Dauwels J, Thalmann N, Zheng J, Maniam Y, Amirah N, Tan BL, Lee J. Prediction of Negative Symptoms of Schizophrenia from Emotion Related Low-Level Speech Signals. 2018 IEEE Int Conf Acoust Speech Signal Process ICASSP Calgary, AB: IEEE; 2018. p. 6024–6028. doi: 10.1109/ICASSP.2018.8462102

13. Stepanov E, Lathuiliere S, Chowdhury SA, Ghosh A, Vieriu R-L, Sebe N, Riccardi G. Depression Severity Estimation from Multiple Modalities. arXiv; 2017. doi: 10.48550/arXiv.1711.06095

14. Yang T-H, Wu C-H, Huang K-Y, Su M-H. Detection of mood disorder using speech emotion profiles and LSTM. 2016 10th Int Symp Chin Spok Lang Process ISCSLP Tianjin, China: IEEE; 2016. p. 1–5. doi: 10.1109/ISCSLP.2016.7918439
